# Conditional Dependence across Slow and Fast Item Responses: With a Latent Space Item Response Modeling Approach

**DOI:** 10.3390/jintelligence12020023

**Published:** 2024-02-16

**Authors:** Nana Kim, Minjeong Jeon, Ivailo Partchev

**Affiliations:** 1 Department of Educational Psychology, College of Education and Human Development, University of Minnesota, Twin-Cities, MN 55455, USA; 2 Social Research Methodology, Department of Education, School of Education and Information Studies, University of California, Los Angeles, CA 90095, USA; mjjeon@ucla.edu; 3 Cito, 6814 CM Arnhem, The Netherlands; partchev@gmail.com

**Keywords:** latent space item response models, conditional dependence, response times, item and person specificities, response process, cognitive tests

## Abstract

There recently have been many studies examining conditional dependence between response accuracy and response times in cognitive tests. While most previous research has focused on revealing a general pattern of conditional dependence for all respondents and items, it is plausible that the pattern may vary across respondents and items. In this paper, we attend to its potential heterogeneity and examine the item and person specificities involved in the conditional dependence between item responses and response times. To this end, we use a latent space item response theory (LSIRT) approach with an interaction map that visualizes conditional dependence in response data in the form of item–respondent interactions. We incorporate response time information into the interaction map by applying LSIRT models to slow and fast item responses. Through empirical illustrations with three cognitive test datasets, we confirm the presence and patterns of conditional dependence between item responses and response times, a result consistent with previous studies. Our results further illustrate the heterogeneity in the conditional dependence across respondents, which provides insights into understanding individuals’ underlying item-solving processes in cognitive tests. Some practical implications of the results and the use of interaction maps in cognitive tests are discussed.

## 1. Introduction

### 1.1. Background

In recent years, there has been increased attention to the use of response times as an additional source of information to improve the estimation of construct(s) or understanding of the underlying response processes in cognitive assessments (Bolsinova et al. 2017c; Bolsinova and Tijmstra 2018; De Boeck and Partchev 2012; DiTrapani et al. 2016; Meyer 2010; Molenaar and De Boeck 2018; Ulitzsch et al. 2020; van der Linden and Guo 2008; Wang and Xu 2015; Wang et al. 2018; Wise and DeMars 2006; Wise and Kong 2005). An extensive body of research has explored the relationship between item responses and response times and examined ways to incorporate response times into psychometric models. The earliest, prominent approach is the hierarchical model proposed by van der Linden (2007), which specifies separate models for item responses (i.e., response accuracy) and response times assuming a bivariate normal distribution for person ability and speed parameters. Key assumptions of this approach include three types of conditional independence: (1) responses are conditionally independent given a person’s latent ability, (2) response times are conditionally independent given a person’s latent speed, and (3) responses and response times are conditionally independent given the person’s ability and speed. The last assumption implies that the association between responses and response times on an item should be fully explained by the relationship between the latent variables in the response and response-time models.

Recent studies, however, have illustrated that the conditional dependence between item responses and response times often exists. That is, there is residual dependence between responses and response times that cannot be accounted for by person and item parameters and their associations. Several studies implementing different methodological approaches to cognitive test data have consistently shown that response accuracy and response times tend to have a negative residual dependency (i.e., shorter response times are associated with higher accuracy) (Bolsinova et al. 2017a, 2017c; DiTrapani et al. 2016; Goldhammer et al. 2014; Molenaar and De Boeck 2018; Partchev and De Boeck 2012) and that such dependency is positively correlated with item difficulty, suggesting that the negative dependency is stronger for easier items while the dependency becomes weaker or even positive for difficult items (Bolsinova et al. 2017a, 2017c; Goldhammer et al. 2014; Meng et al. 2015; Molenaar and De Boeck 2018; see De Boeck and Jeon 2019). Such findings suggest that a respondent’s response accuracy tends to increase with shorter response times for easy items whereas the accuracy increases relatively less or even decreases with faster responses for difficult items.

While most existing research focuses on exploring and explaining the general pattern of residual dependency between item response and response times that can apply to all respondents and items, it is plausible that the same pattern is not applicable to every individual and item. For instance, it is known that easy items are associated with more successful responses when responses are given faster; however, this may not hold for certain respondents and/or items as some respondents may find certain easy items more difficult with faster responses. Similarly, although it has been shown that the response accuracy for difficult items may tend to increase with slower responses, it may not be true for some respondents. As a result, the nature of conditional dependence between item responses and response times can be more complex than we think, involving person and item specificities. In this respect, the current study aims to explore the complexity and specificity of the residual dependency between responses and response times by examining how respondents and items exhibit heterogeneous residual associations between responses and response times.

### 1.2. Current Study

We employ a latent space item response theory (LSIRT; Jeon et al. 2021), a recently proposed approach for modeling the residual dependence of item responses, to explore the conditional dependence between item responses and response times. The LSIRT model assumes that respondents and items are embedded in a latent space and that the probability of a correct response decreases as the distance between the positions of a respondent and an item increases given the respondent’s ability and the item’s difficulty. That is, the item-by-person distances represent interactions between respondents and items that are not explained by the respondent’s and item’s main effects (the person’s ability and item difficulty), i.e., conditional dependence between respondents and items. An important advantage of the LSIRT is that it visualizes conditional dependence between respondents and items in a low-dimensional Euclidean space called an interaction map. Inspecting an interaction map can help us evaluate how individual respondents interact with specific items, e.g., which items they are stronger or weaker with given their ability levels. Such information can be used to formulate individualized diagnostic feedback for the respondents.

We incorporate the item response time information into the LSIRT model, borrowing the idea of a response time classification approach from previous studies (e.g., DiTrapani et al. 2016; Molenaar and De Boeck 2018; Partchev and De Boeck 2012). These studies examined conditional dependence between responses and response times by classifying each item-by-respondent response into slow or fast, inspecting systematic differences in item characteristics across slow and fast responses. Leveraging this idea, we classify item responses into slow and fast based on response times and then fit the LSIRT model to estimate separate latent positions for slow and fast responses for an item. Our intent is to demonstrate the potential presence of conditional dependence between item responses and response times and its heterogeneity, not to propose an alternative approach for jointly modeling item-response times and item responses.

It is worth noting that we use the *residual* log-transformed item-response times (after accounting for person and item effects) for classification. This is to examine the residual/conditional dependence between responses and response times not attributable to person and item effects. A comparison of latent positions across slow and fast responses then provides information on how respondents are expected to behave or interact with an item differently depending on whether they respond faster or slower than expected, shedding light on the residual dependency between responses and response times. Such information can offer insights into understanding individuals’ item-solving processes for cognitive test items, providing personalized diagnostic feedback for each respondent. For example, our analysis may identify that a respondent is better on a certain type of item when responding faster, while others do not show such a pattern.

Our work is distinguished from recent work that examined the heterogeneity in conditional dependence between responses and response times (Kang et al. 2022b, 2023). These studies explored varying conditional dependence across items and respondents based on a diffusion IRT modeling framework (Tuerlinckx and De Boeck 2005; Tuerlinckx et al. 2016; van der Maas et al. 2011). They specifically introduced the variability in the drift rate and starting point of the decision process to the model and interpreted the variability in these parameters as the heterogeneity in the conditional dependence (Kang et al. 2022b). Kang et al. (2023) further applied a latent space modeling approach with a diffusion IRT model, assuming that the item–respondent distance in an interaction map can capture the random variability in the drift rate.

In contrast, our approach applies a latent space modeling approach with a standard IRT model for item responses where the distance term directly explains the correct response probability. Importantly, we represent item responses and response time information (slow and fast) on an interaction map simultaneously, facilitating an intuitive evaluation of the varying item–respondent interactions at different speeds. We believe these features enable an easier interpretation of the interaction map and, thus, a straightforward examination of the conditional dependence between responses and response times.

### 1.3. Structure

The remainder of this paper is organized as follows. In the following section, we describe the latent-space item-response theory (LSIRT) model. In Section 3, we describe our approach in terms of the way we classify each item-by-respondent response into fast or slow, and how we fit the model to estimate the latent positions of slow and fast responses separately for each item. Followed by the descriptions of empirical datasets and how they are analyzed in Section 4, we illustrate the heterogeneity in item–respondent interactions across slow and fast responses through an examination of interaction maps derived from the model. We further demonstrate the residual dependency captured by the interaction map even after accounting for differences in item difficulty across fast and slow responses. We conclude with a discussion of the results and future directions for research.

## 2. Latent-Space Item-Response Theory

A core assumption of traditional item-response theory (IRT) models is that item responses are conditionally independent given person and item parameters (i.e., a local/conditional independence assumption). However, this assumption can often be violated in practice, and various approaches have been developed and used to address such violations (e.g., testlet and bifactor models and finite mixture models). Jeon et al. (2021) recently proposed a novel modeling approach, a latent-space item-response theory (LSIRT), to account for conditional dependence among item responses. Compared with existing approaches, the LSIRT provides a flexible tool to attend to unobserved heterogeneity across item responses without any prior knowledge and to explore residual item–respondent interactions unexplained by item and person parameters in a graphical way.

In this paper, we consider a Rasch version of the LSIRT model. The conventional Rasch model (Rasch 1980) assumes that the probability of item correctness is characterized by two main parameters: item easiness (*c*) and the person’s ability (θ). Thus, the Rasch model specifies the log odds of the probability of a correct response as

(1) $$logit\left(P\left(Y_{pi}=1|\theta_{p},c_{i}\right)\right)=\theta_{p}+c_{i},$$

where $Y_{pi}=1$ is a correct response of person p(=1,⋯,P) on item i(=1,⋯,I), $\theta_{p}$ is a latent proficiency parameter of person *p*, and $c_{i}$ is an item-easiness parameter of item *i*. To account for the item–respondent interactions that remain after explaining by $\theta_{p}$ and $c_{i}$, the LSIRT introduces an item–person specific distance term to the traditional model above. Specifically, the LSIRT assumes that persons and items are located in a common latent space called an interaction map, and the probability of a correct response decreases as the distance between a person and an item increases. Under these assumptions, the LSIRT model is specified as

(2) $$logit\left(P\left(Y_{pi}=1|\theta_{p},c_{i},a_{p},b_{i}\right)\right)=\theta_{p}+c_{i}-\gamma \cdot d\left(a_{p},b_{i}\right),$$

where $a_{p}$ and $b_{i}$ represent position vectors of the respondent *p* and item *i*, respectively, in the interaction map; $d(a_{p},b_{i})$ is the distance between $a_{p}$ and $b_{i}$; and the weight γ≥0 indicates the effect of the distance term on the correct response probability. Among several distance functions, we consider Euclidean distance defining $d\left(a_{p},b_{i}\right)=\sqrt{\Sigma_{j=1}^{k}{\left(a_{pj}-b_{ij}\right)}^{2}}$ in a *k*-dimensional space. By incorporating this distance term into the model, the LSIRT model is able to account for interactions between items and persons, i.e., conditional (or residual) dependence among item responses, which is not explained by the person and item parameters. We constrain γ to be larger than 0 so as to force the negative relationship between the distance and the probability of a correct response. This implies that a larger distance between a person and an item leads to a lower probability of item correctness than the expected probability from the person and item parameters. When γ=0, the LSIRT model (Equation (2)) reduces to the conventional Rasch model (Equation (1)).

**Figure 1:**
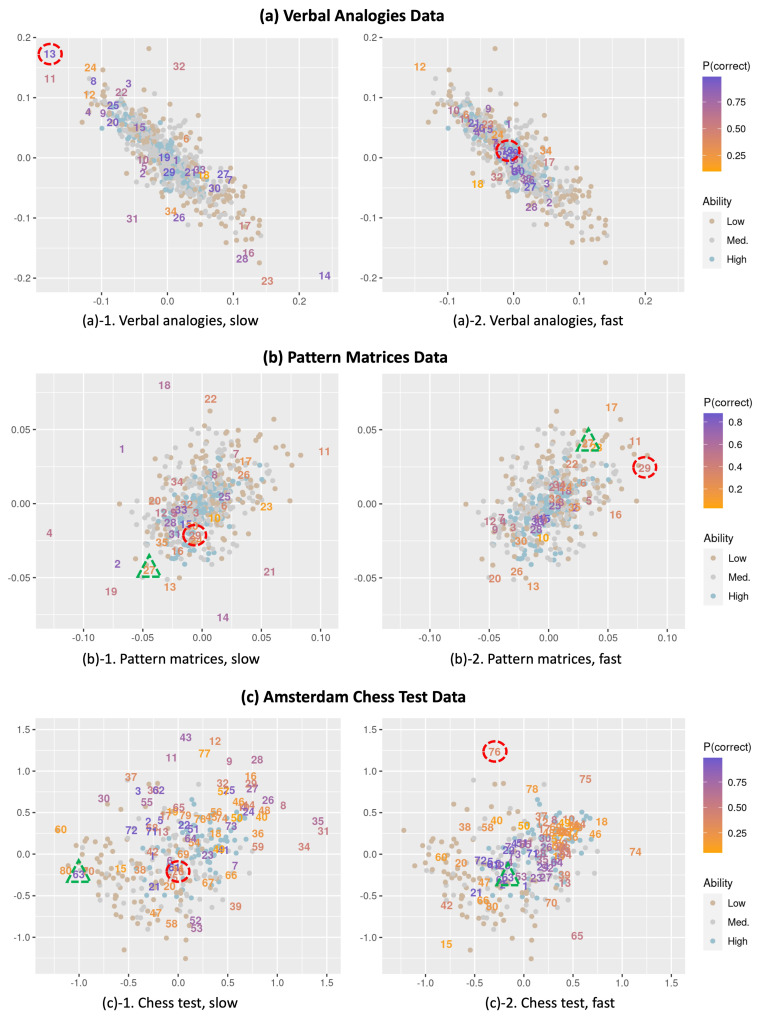
Item–respondent interaction maps for items under slow- and fast-response conditions, derived from latent-space item-response models constraining item parameters to be equal across slow and fast responses for the same item for (**a**) verbal analogies, (**b**) pattern matrices, and (**c**) Amsterdam chess test data.

**Figure 4:**
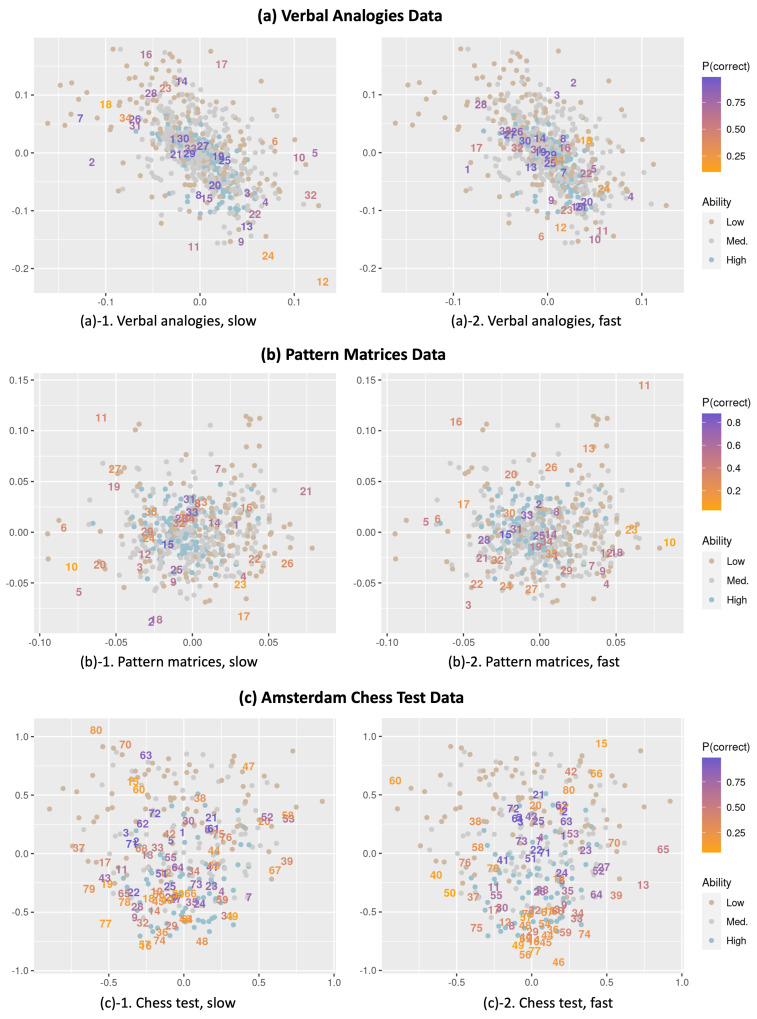
Item–respondent interaction maps for items under slow- and fast-response conditions, derived from latent-space item-response models allowing for a separate estimation of item parameters for slow and fast responses for the same item for (**a**) verbal analogies, (**b**) pattern matrices, and (**c**) Amsterdam chess test data.

A powerful feature of the LSIRT is that it supplies an interaction map, which is a graphical representation of the residual dependence of persons and items (e.g., Figures 1 and 4 in this paper; Figure 1 in Jeon et al. 2021). The interaction map is obtained by mapping the estimated positions of persons and items (i.e., ${\hat{a}}_{p}$, ${\hat{b}}_{i}$ ) onto a low-dimensional space (often a two-dimensional space, i.e., k=2). We note that the LSIRT model is closely related to multidimensional scaling (MDS) and can be viewed as a model-based MDS (Hoff et al. 2002). Similar to MDS, the person and item positions in the interaction map represent how each person and/or item relates to each other. For example, similar items or persons would tend to show shorter distances between their positions, and a shorter distance between a person and an item would indicate that the person is likely to show a higher probability of getting the item correct than what is expected from their ability and item difficulty. As can be inferred from Equation (2), the interaction map captures the deviations from the person and item parameters (i.e., the main effects) of the model, effectively reflecting conditional dependence among item responses due to the unobserved heterogeneity of persons and items. Hence, inspecting an interaction map can help us improve our understanding of interactions between persons and items, i.e., how item(s) may function differently for different respondents.

## 3. Our Approach

We aim to explore how individuals interact with items differently depending on their response times by looking at the interaction maps for slow and fast item responses through the LSIRT approach. To this end, we separate item-by-respondent responses into slow and fast based on whether a response is slower or faster than what is expected from the person’s speed and item intensity. We then produce an item–respondent interaction map by estimating separate positions for slow and fast responses for an item within the LSIRT framework. Effectively, we treat slow and fast responses to the same item as though they are different items to identify the positions for slow and fast responses independently. Below we describe in detail (1) how we classify item responses into slow and fast responses and (2) how we identify the latent positions of slow and fast responses for an item.

### 3.1. Classification of Item Responses into Slow and Fast Responses

For the dataset under investigation, we classify each item-by-respondent response ($Y_{pi}$) into slow or fast based on whether the response is given slower or faster than the expected response times given the person’s speed and item intensity. Specifically, we estimate the residual response times by removing the person and item effects (i.e., we obtain double-centered response times) and then classify the item response into slow if the residual response time is positive and fast if negative. We use a cross-classified random effects model to estimate the residual response times, treating the item-response times as nested within both persons and items. The model specifically characterizes response times ($RT_{pi}$) as

(3) $$\begin{array}{c} \ln\left(RT_{pi}\right)=\beta_{0}+u_{p}+u_{i}+e_{pi}, \end{array}$$

where $\beta_{0}$ is the model intercept representing the grand mean; $u_{p}∼N\left(0,\sigma_{u_{1}}^{2}\right)$ and $u_{i}∼N\left(0,\sigma_{u_{2}}^{2}\right)$ denote the person (*p*) and item (*i*) random effects, respectively; and $e_{pi}∼N\left(0,\sigma_{e}^{2}\right)$ is the residual for a person *p* and item *i* (i.e., the double-centered response times). As the person and item random effects explain the person’s speed and item intensity, respectively, the residual term $e_{pi}$ represents the deviation from the expected log response times for the person to respond to the item. Note that we log transform the response times (i.e., $\ln(RT_{pi})$) due to its skewness. We use the *lme4* package (Bates et al. 2015) in R (R Core Team 2022) to fit the model and obtain the residual log response time $e_{pi}$.

We define an item-by-respondent response ($Y_{pi}$) as slow if its corresponding residual log response time is greater than or equal to 0 ($e_{pi}\geq 0$) and fast if below 0 ($e_{pi}<0$), as aforementioned. Because the person and item random effects in Equation (3) already account for the response times due to person and item attributes (e.g., the person’s cognitive processing speed and item complexity), the classification based on the residual term allows each person and item to have relatively fast and slow responses irrespective of the person’s speed and item features. Thus, slow and fast responses in this study imply that the responses are given relatively slower and faster than what is expected from the person and item, rather than indicating the absolute sense of the response speed. The use of residual item-response times for classification allows us to examine the *residual* dependency between responses and response times and a *within-person* heterogeneity in item–respondent interactions across response times (Bolsinova et al. 2017c; Kim and Bolt 2023; Molenaar and De Boeck 2018). It also produces a balance of both persons and items in fast and slow conditions, making it more feasible to compare item parameters and interaction maps across the two conditions. We provide the R codes used to estimate the residual log response times and classify item responses into slow and fast conditions in https://osf.io/jvg7u/?view_only=e03fae69923842df8da364224a3a2e51 (can be accessed on 10 February 2024).

### 3.2. Estimation of Separate Latent Positions of Slow and Fast Responses for an Item

Once we classify item responses into a slow or fast condition by their associated response times, we consider the data as mixtures of slow and fast responses. Because we are interested in looking at how item–person interactions vary depending on whether an item response is given faster or slower, we estimate separate latent positions (to be mapped onto the interaction map of the LSIRT model) for the slow and fast conditions of an item. To do so, we treat fast and slow responses to the same item as if they are responses to different items and create an expanded item-response matrix by combining two separate item-response matrices for slow- and fast-response conditions (as illustrated in Table 1). The expanded matrix has a double number of items (2×I) in which fast responses are treated as missing (NA) under the slow-response condition and vice versa. Because each one of respondent’s item responses is either in the fast or slow condition, the resulting data structure has exactly half of the item responses missing for each respondent. By fitting the LSIRT model to such expanded data, we can simultaneously fit the model to both slow and fast responses and estimate independent latent positions for slow and fast conditions for each item. This approach is similar to what is performed in typical item-response tree (IRTree) models to estimate separate model parameters for sibling nodes (Böckenholt 2012; De Boeck and Partchev 2012; Jeon and De Boeck 2016) (can be accessed on 10 February 2024).

We consider two LSIRT models for data analysis:

- The constrained model—same item easiness across slow and fast responses for an item: We constrain the item parameter $c_{i}$ (in Equation (2)) to be equal across fast and slow conditions. That is, we constrain $c_{i}$ to be equal to $c_{I+i}$ so that each item has a single easiness parameter regardless of the response-speed conditions. Such a constraint will allow the interaction map to capture the heterogeneity in the item–respondent interactions across response times that may be due to either items or persons or both. For instance, the distances we observe from the interaction map may reflect how each item performs differently depending on whether it is answered faster or slower by respondents and how individuals behave differently on different items depending on response times.
- The unconstrained model—separate item easiness across slow and fast responses for an item: We allow for a separate estimation of the item-easiness parameters for slow and fast conditions to examine the remaining heterogeneity after accounting for the differences in item properties across slow and fast responses. This approach removes the heterogeneity across slow and fast responses that is consistent across respondents from the interaction map and helps us explore the heterogeneity that cannot be explained by the items themselves by inspecting the interaction map. Thus, we anticipate that the former model (i.e., the constrained model) captures the heterogeneity in item–person interactions due to the differences in item properties across fast and slow responses as a part of residual dependence, whereas the latter model (i.e., the unconstrained model) captures any potential heterogeneity that remains even after accounting for the item-difficulty differences across response times.

## 4. Empirical Analysis

We analyze three different empirical datasets from the verbal analogies, pattern matrices, and Amsterdam chess tests. All three tests are designed to measure cognitive abilities; the first two tests are inductive reasoning intelligence tests and the last one measures chess intelligence. Both the verbal analogy and pattern matrices data were used by DiTrapani et al. (2016); Kang et al. (2022a, 2023); Partchev and De Boeck (2012) and the chess data were introduced by van der Maas and Wagenmakers (2005). More detailed descriptions of each dataset are provided below.

### 4.1. Data

#### 4.1.1. Verbal Analogies

Verbal analogies are considered a common type of intelligence test measuring general intelligence as well as crystallized intelligence largely determined by the level of word knowledge (Bejar et al. 1991; Levine 1950; Ullstadius et al. 2008). For the current analysis, we use a subset of the data from calibration studies for computerized verbal analogies tests developed by Hornke (Hornke 1999, 2002; Hornke and Rettig 1993. The original complete dataset contains the item responses (the item correctness) and their response times (in seconds) for 254 items from 25 test forms (of 24 items each) with partially overlapping items. We used the same subset of data used in Partchev and De Boeck (2012) containing responses from 726 respondents to 34 items. Due to the block design, each respondent responded to 10 or 12 items among the 34 items and had systematic missing responses. The test was administered with a time limit of 180 s per item and, thus, we remove item responses with response times longer than 180 s (n=5), considering them as invalid responses. More details on the test and/or the data can be found in DiTrapani et al. (2016); Partchev and De Boeck (2012); Partchev et al. (2013).

#### 4.1.2. Pattern Matrices

Matrices are another popular type of intelligence test tapping on inductive reasoning. The test we used was developed by Hornke (Hornke 2002; Hornke and Habon 1986) and consists of multiple-choice items with eight response options. We use the same subset of data analyzed by DiTrapani et al. (2016); Kang, De Boeck, and Partchev (2022a); Kang et al. (2023); Partchev and De Boeck (2012), which has the item responses (the item correctness) and response times (in seconds) from 504 respondents to 35 items. This test also has multiple forms administered with a block design, so each respondent responded to a subset of the items. The test had a time limit of 180 s per item. We eliminate invalid item responses with response times longer than 180 s (n=239;4% of the total responses) in addition to responses from a potentially inattentive respondent who spent less than 5 s for every item. Consequently, the final dataset contains item responses and response times from 503 respondents to 35 items with systematic missing data. See DiTrapani et al. (2016); Partchev and De Boeck (2012) and Hornke and Habon (1986) for more detailed descriptions of the test and/or data.

#### 4.1.3. Amsterdam Chess Test

The Amsterdam chess test (ACT) introduced by van der Maas and Wagenmakers (2005) is a computerized test measuring chess expertise or proficiency. We use the data that come from the choose-a-move test consisting of information-processing tasks (i.e., reasoning tasks) among several subsets of the ACT. This test involves chess problems asking respondents to find the single best move from a presented chess diagram as quickly as possible. The choose-a-move test has two parallel test forms (A and B), and each form consists of 40 items concerning three different item domains: 20 tactical items, 10 positional items, and 10 endgame items. While the three item types each measure specific chess abilities/knowledge (i.e., common tactics, positional judgment, and endgame knowledge), they collectively measure general chess proficiency (van der Maas and Wagenmakers 2005). Each item clearly has the best move among many possible moves; thus, responses were scored correct if a person correctly made the best move and incorrect otherwise. There was a time limit of 30s per item. The original data have responses and response times from 259 respondents to 80 items (40 items for each test form). Different from the two previous datasets, all 80 items were administered to all respondents. However, 10 respondents did not take either test A or B, or both, so they were excluded from the final dataset. Consequently, the final data analyzed in this study contain responses from 249 respondents.

### 4.2. Analytic Methods

As described in Section 3.2, we fit two LSIRT models to each dataset with slow and fast responses separated as different item responses: (1) constrained and (2) unconstrained models. The constrained model sets the item-easiness parameters to be equal across fast and slow conditions for the same item, while the unconstrained model allows them to vary. Essentially, the interaction maps derived from the two models capture residual dependency from slightly different sources; the constrained model captures all the potential sources including the item differences across response times, whereas the unconstrained model captures sources unexplained by the item differences.

#### 4.2.1. Model Estimation

The LSIRT models are estimated by using a fully Bayesian Markov chain Monte Carlo (MCMC) method. Specifically, we implement the No-U-Turn Sampler (NUTS) (Hoffman and Gelman 2014) in Stan (Stan Development Team 2023b). NUTS is an extension of the Hamiltonian Monte Carlo algorithm that enables an efficient estimation of complex models without hand tuning. We use the ‘RStan’ package (Stan Development Team 2023a) in R (R Core Team 2022) to run Stan models. The R codes we used to fit the models are available at https://osf.io/jvg7u/?view_only=e03fae69923842df8da364224a3a2e51 (can be accessed on 10 February 2024).

The prior distributions specified for the model parameters of the LSIRT are as follows:

$$\begin{array}{c} \theta_{p}∼N\left(0,\sigma^{2}\right),\sigma^{2}\geq 0\\ c_{i}∼N\left(\mu_{c},\sigma_{c}^{2}\right),\sigma_{c}^{2}\geq 0\\ \gamma ∼N\left(0,3^{2}\right)I\left(0,\right)\\ a_{p}∼MVN_{k}\left(0,I_{k}\right)\\ b_{i}∼MVN_{k}\left(0,I_{k}\right), \end{array}$$

where I(0,) indicates that γ is constrained to be a positive value; $MVN_{k}$ denotes a *k*-dimensional multivariate normal distribution; and 0 and $I_{k}$, respectively, are the *k*-dimensional zero vector and identity matrix. Note that we impose priors on the coordinates of items and persons instead of the distances as it is more straightforward (Jeon et al. 2021). In this study, the number of dimensions of the latent space is considered to be k=2 for easier interpretation and visualization of the latent space. As for the hyperparameters, we assume a $N(0,5^{2})$ prior for $\mu_{c}$ and half-Cauchy priors with a location of 0 and scale of 5 for σ and $\sigma_{c}$.

#### 4.2.2. Model Identifiability

An important aspect to consider for the LSIRT model is identifiability. Because the distance between the positions of a person and item in the latent space (i.e., $d(a_{p},b_{i})$) is invariant under rotations, translations, and reflections, an infinite number of positions can result in the same distance and, consequently, the same likelihood value (Hoff et al. 2002; Jeon et al. 2021; Jin and Jeon 2019; Oh and Raftery 2007; Shortreed et al. 2006). To address this nonidentifiability issue, we postprocess the posterior samples by using Procrustes matching (Borg and Groenen 2005, Chapter 20), as was performed in previous latent-space-modeling approaches (Friel et al. 2016; Hoff et al. 2002; Jeon et al. 2021; Jin and Jeon 2019; Shortreed et al. 2006). We implement the procedure described and used in Friel et al. (2016); Jin and Jeon (2019). Specifically, we take the latent coordinates from the iteration with the highest log posterior density as a reference set (or the target configuration) and apply a Procrustes transformation to each of all the other posterior samples so as to approximate the target configuration as closely as possible. The relevant code is available in the ‘prolsirm’ package in R (Luo and Jeon 2023).

#### 4.2.3. Model Evaluation

We simultaneously run two independent chains of 10,000 iterations each and then discard the first half of the iterations for the warm up. Thus, the posterior distributions are approximated by using 10,000 posterior samples (=5000 iterations × 2 chains). To evaluate the model convergence, we examine the potential scale-reduction factor (PSRF or $R^{2}$) values (Gelman and Rubin 1992) for all the model parameters and visually inspect the traceplots of the post-warm-up samples. Note that we evaluate the convergence of the distance measures between the positions of persons and items rather than the coordinates themselves. To assess the absolute model fit, we use posterior predictive checking (PPC) to examine the discrepancy between the observed and generated data from the posterior predictive distribution (Gelman et al. 2013, Chapter 6). We specifically compare the observed and posterior predictive proportions of correct responses at the item level and the respondents’ total score distributions.

**Figure A1:**
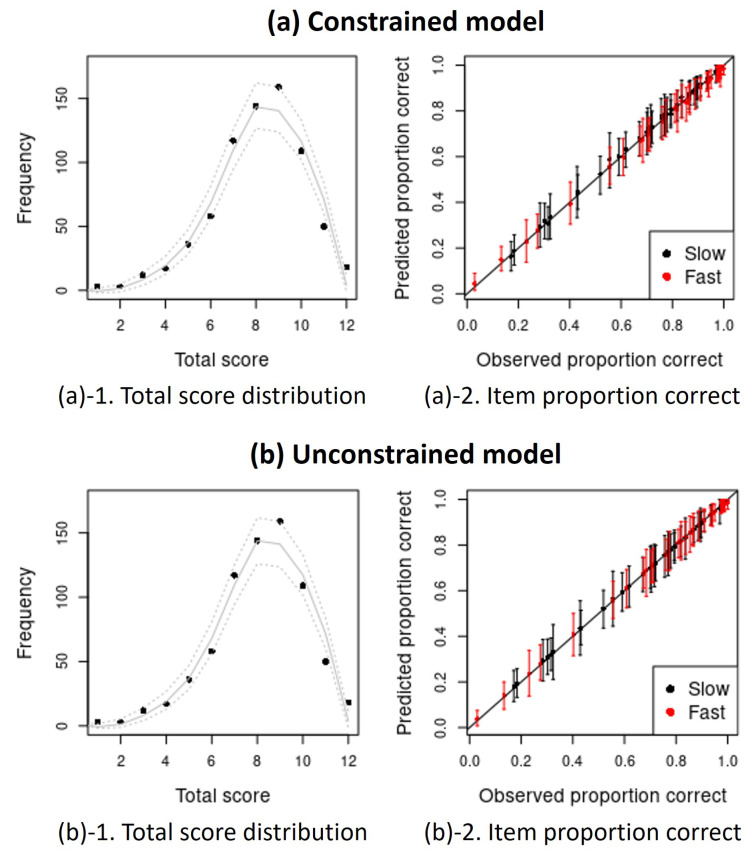
Posterior predictive checks (PPCs) plots for verbal analogies data: (**a**) constrained and (**b**) unconstrained models. For total score distribution plots, solid circles represent the observed data, solid gray lines indicate the 50th percentile, and dotted gray lines represent the 5th and 95th percentiles of the generated samples. For proportion of correct items plots, solid circles indicate the observed data, and error bars show the 5th and 95th percentiles of the generated samples.

**Figure A2:**
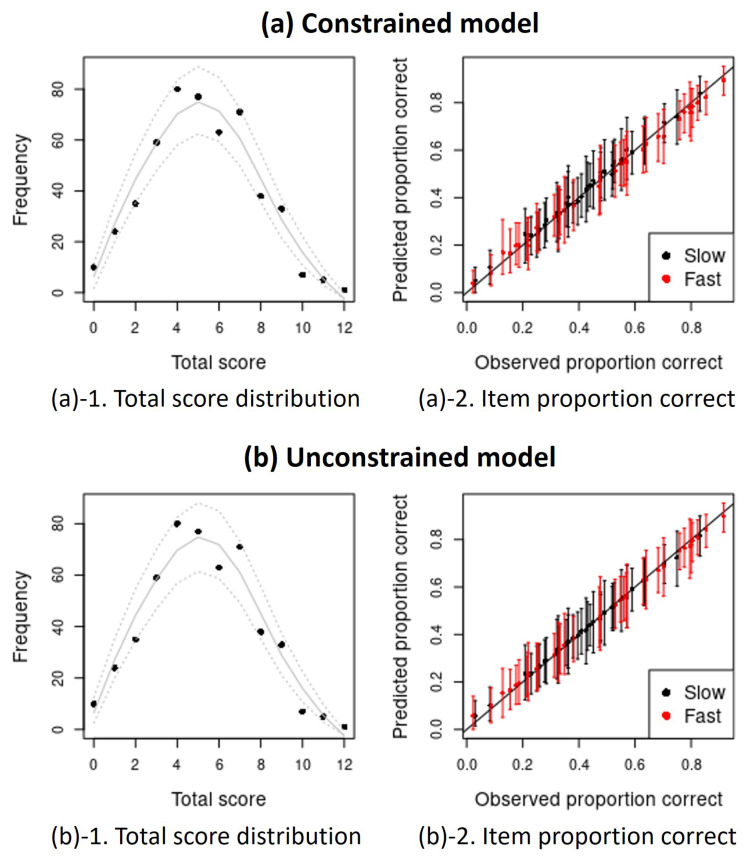
Posterior predictive checks (PPCs) plots for pattern matrices data: (**a**) constrained and (**b**) unconstrained models. For total score distribution plots, solid circles represent the observed data, solid gray lines indicate the 50th percentile, and dotted gray lines represent the 5th and 95th percentiles of the generated samples. For proportion of correct items plots, solid circles indicate the observed data and error bars show the 5th and 95th percentiles of the generated samples.

**Figure A3:**
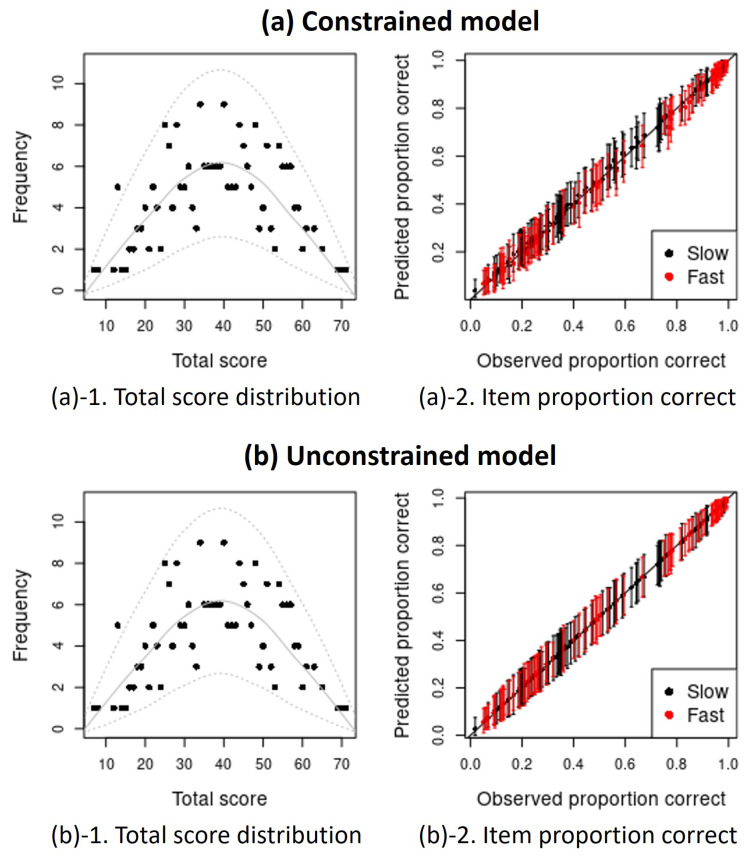
Posterior predictive checks (PPCs) plots for ACT data: (**a**) constrained and (**b**) unconstrained models. For total score distribution plots, solid circles represent the observed data, solid gray lines indicate the 50th percentile, and dotted gray lines represent the 5th and 95th percentiles of the generated samples. For proportion of correct items plots, solid circles indicate the observed data and error bars show the 5th and 95th percentiles of the generated samples.

Across all datasets and models, the PSRF values (Gelman and Rubin 1992) for all parameters are below 1.05, and the traceplots show well-mixed and caterpillar-like shapes, suggesting satisfactory model convergence. The results of posterior predictive checks also indicate that the two models can sufficiently explain the data at both item and respondent levels (see Figure A1, Figure A2 and Figure A3 in Appendix A). Based on the two models fitted, we compare the interaction maps across slow and fast responses to examine residual dependency between responses and response times.

## 5. Results

### 5.1. Constrained Model: Heterogeneity of Item–Respondent Interactions across Slow and Fast Responses

We first inspect the results based on the LSIRT model constraining the item parameters to be equal across the slow- and fast-response conditions for the same item. For all three datasets, the effects of the distance term (γ) in the LSIRT are larger than 0, indicating that there is significant residual dependence among items and respondents that cannot be explained by the main person and item parameters in the model. Specifically, the estimates for γ are 1.491 [1.238, 1.751], 1.148 [.863, 1.423], and 1.164 [1.047, 1.291] for the verbal analogies, pattern matrices, and ACT data, respectively.

We examine the latent interaction map derived from the constrained model for a close inspection of the residual dependence in each dataset. Of particular interest would be the heterogeneity of the item–respondent interactions across the fast and slow item-response conditions. For an easier comparison across the slow and fast responses, we plot the items for slow and fast conditions in separate interaction maps, as shown in Figure 1. The following points can be helpful to understand Figure 1.

- We present the interaction map for the slow- and fast-response conditions separately for ease of interpretation. Note that the positions from the two maps are obtained from a single model for the same dataset. Therefore, the positions of the respondents (represented as dots) are the same in the two maps.
- Items are represented as their original item number for both the slow and fast conditions (instead of the item numbers in the expanded data format) so that the item positions for slow and fast responses can be easily compared. Thus, for example, by comparing the positions for the same item number in the interaction maps for the slow- and fast-response conditions, we can observe how an item behaves differently depending on whether it was responded to slower or faster than expected.
- We color the respondents and items each based on their levels of ability estimates and the proportion that each item is correct (the overall item easiness). We define the respondents’ ability as low, medium, and high if the estimates are below −.5, between −.5 and .5, and above .5, respectively, and each group is colored differently. There are 162, 377, and 187 respondents each in the low, medium, and high groups for the verbal analogies data, respectively; 137, 240, and 126 respondents for the matrices data, respectively; and 83, 91, and 75 respondents for the ACT data, respectively. For the items, easier items (with a higher proportion correct) are colored purple while more difficult items (with a lower proportion correct) are colored orange.

**Items with slow responses are farther away from respondents.** One common observation across the three datasets is that item positions are generally more scattered under the slow-response condition than the fast-response condition. Such a result possibly indicates that items tend to show more heterogeneity in item–person interactions when responded to slower than expected, while the interaction is less heterogeneous when responded to faster. As items for slow responses are more spread out, we can naturally expect slow responses, on average, to have a greater distance from the positions of respondents compared to fast responses.

**Figure 2:**
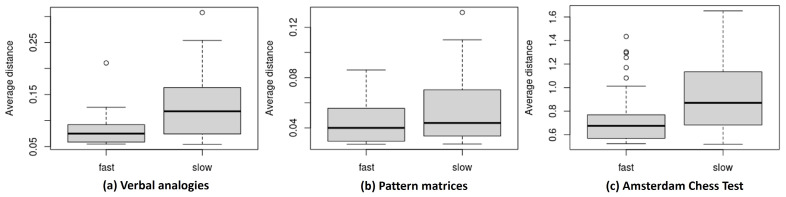
Boxplots of items’ average distance from individuals for slow- and fast-response conditions for (**a**) verbal analogies, (**b**) pattern matrices, and (**c**) Amsterdam chess test data.

Figure 2 illustrates the ranges of the mean item–respondent distances of the items (${\bar{d}}_{i}$), calculated by averaging the Euclidean distance over the respondents per item for the slow and fast responses, where ${\bar{d}}_{i}=\Sigma_{p=1}^{P}d\left(a_{p},b_{i}\right)/P$. The comparison of the boxplots of the item distance between the fast and slow responses suggests that items under the slow condition are, on average, located farther away from the respondents. Recalling that a larger distance is associated with a lower probability of getting an item correct than what is expected, such an observation can be interpreted as meaning that the items are generally more difficult (associated with a lower accuracy) when the responses are given slower. In other words, faster responses can more likely lead to successful responses. As an example,

- Item 13 in the verbal analogies data (a relatively easy item located at the top left of plot (a)-1 and in the middle of plot (a)-2 in Figure 1), the proportion correct is .865 for the slow responses (of which the average item–respondent distance is .251) and .987 for faster responses (the average distance is .056).
- Likewise, Item 63 in the ACT data (located on the left side of plot (c)-1 and toward the center of plot (c)-2) produces a relatively lower proportion correct (.820) for the slow responses (average distance = 1.002) than for the fast responses (proportion correct = .949, average distance = .544).

**The pattern of association varies by the overall item difficulty.** The observed pattern above is particularly apparent and consistent for easier items (purple-colored items) while some relatively difficult items (orange-colored items) show a weak or even an opposite pattern. For example,

- Item 29 with a moderate difficulty (overall proportion correct = .404) in the pattern matrices data is located inside the item cluster around the middle of plot (b)-1 and in the upper right of plot (b)-2 in Figure 1, showing a higher proportion correct (.519) for the slow responses (average distance = 0.033) and a lower proportion correct (.250) for the fast responses (average distance = .086).
- Item 76 in the ACT data, which is relatively difficult (overall proportion correct = .378) and located toward the center of plot (c)-1 and at the top of plot (c)-2 in Figure 1, appears to be easier for the slow responses (proportion correct = .566, average distance = .527) and more difficult for the fast responses (proportion correct = .150, average distance = 1.434).

Such findings align with previous results on local dependence between RT and accuracy, which have demonstrated that easier items tend to have a negative dependency (i.e., fast responses have higher accuracy), and this association becomes weaker or even positive (e.g., slow responses have higher accuracy) for relatively more difficult items (Bolsinova et al. 2017a, 2017c; DiTrapani et al. 2016; Goldhammer et al. 2014; Meng et al. 2015; Molenaar and De Boeck 2018; Partchev and De Boeck 2012).

**Item–respondent interactions vary across respondents.** While we observe differences in item difficulty across response times, it is important to highlight that such a pattern only explains a part of the residual dependency between responses and response times captured in the interaction map. Every individual interacts with items in different ways, and thus the relationship between responses and response times may not be the same for all respondents and items. For instance, an item that is easier when responded to faster for some respondents can be easier when responded to slower for other respondents and vice versa. Such heterogeneity can be examined by looking at the positions of individual respondents and items in the interaction map across fast and slow responses. The heterogeneity is expected to be more apparently demonstrated by items exhibiting very different positions in the interaction map across fast and slow responses. For example,

- Although Item 63 in the ACT data is shown to be generally easier for fast responses (as described above), respondents who are located around the position of Item 63 in plot (c)-1 of Figure 1 would show a lower accuracy for fast responses (as manifested by larger distances between these individuals and Item 63 in plot (c)-2).
- Item 27 in the pattern matrices data is located at the lower left part of plot (b)-1 and the upper right part of plot (b)-2 in Figure 1, which possibly suggests that respondents with positions around the lower left are more likely to get the item correct when responding slower than faster whereas those located around the upper right tend to have a higher probability of getting the item correct when responding faster.

One drawback of interpreting the interaction map under the constrained LSIRT model is that the effect of the item-difficulty differences across response times is confounded with all other potential sources of conditional dependence. In order to facilitate the exploration of heterogeneity in conditional dependence between responses and response times (after removing the item effect), we fit the unconstrained LSIRT model to the data, of which the results will be demonstrated in the following subsection.

### 5.2. Unconstrained Model: Residual Dependency between Item Responses and RT after Accounting for the Heterogeneity in Item Difficulty across Fast and Slow Responses

**Figure 3:**
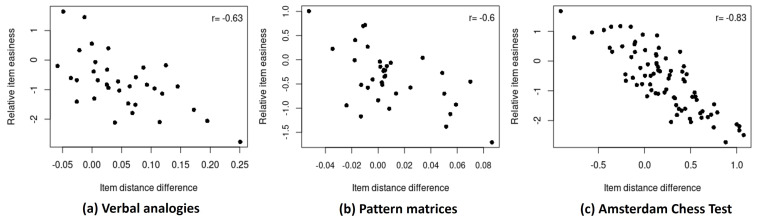
Scatter plots of relative item easiness (under the unconstrained model) against item distance difference (under the constrained model) for (**a**) verbal analogies, (**b**) pattern matrices, and (**c**) Amsterdam chess test data.

The unconstrained model estimates separate item-easiness parameters for the slow- and fast-response conditions of an item, allowing the model parameters to account for the overall differences in the item difficulty across the response times. The estimated item-easiness parameters for the slow and fast responses obtained from the unconstrained model, therefore, are anticipated to have an association with the item’s mean distances observed under the constrained model. Figure 3 presents scatter plots of the difference in the item-easiness estimates across the slow and fast responses of an item (derived from the unconstrained model; $c_{i}-c_{I+i}$) against the difference in the item’s mean distance across the slow and fast responses (derived from the constrained model; ${\bar{d}}_{i}-{\bar{d}}_{I+i}$) for each dataset.

It is shown in Figure 3 that a negative relationship is consistently observed across all three datasets. This indicates that items exhibiting a disproportionately larger mean distance for the slow compared to the fast responses (i.e., a large positive item-distance difference) under the constrained model produce a lower item-easiness estimate for the slow responses than the fast responses (i.e., negative relative item easiness) under the unconstrained model. Such a relationship implies that the differences in the item mean distance across the response times observed in Section 5.1 are sufficiently explained by item-easiness parameters in the unconstrained model. As a result, the distance term in the unconstrained model captures the residual dependency unexplained by the item effects.

The distance effect γ under the unconstrained model is estimated as 1.418 [1.092, 1.709], 1.157 [.250, 1.514], and 1.111 [.979, 1.249] for the verbal analogies, pattern matrices, and ACT data, respectively, suggesting that the conditional dependence is significant even after accounting for the item-difficulty differences across response times.

**More difficult items tend to be more spread out.** As a result of removing the overall item differences in difficulty across the response times from the interaction map, we observe in Figure 4 that the items are now relatively equally spread out across the fast and slow responses compared to Figure 1. In addition, for both the fast and slow responses, the more difficult items (orange-colored) appear generally more spread out in the interaction map than the easier items (purple-colored) that are more clustered. This indicates that the more difficult items tend to show more heterogeneous interactions with respondents that cannot be explained by the person and item parameters, involving a greater amount of residual variation (e.g., item–respondent specificities). A related observation is a larger distance between the slow and fast responses for more difficult items. The distance between the slow and fast responses for an item tends to negatively correlate with the overall item difficulty in all three examples (ranging from −.52 to −.26). This suggests that more difficult items may exhibit a greater heterogeneity in item–respondent interactions across response times.

**The conditional dependence between responses and response times is heterogeneous.** As implied above, some items are located very closely across the fast- and slow-response conditions (e.g., Item 30 in the verbal analogies data) while some other items show a larger distance between the positions for the slow- and fast-response conditions. Such variability suggests that the amount of residual dependency between the responses and response times is different across items. A shorter distance implies that there is not much conditional dependence remaining between the responses and response times; thus, respondents would behave similarly for the slow and fast responses (in a way captured by the item-difficulty differences between the slow and fast responses). In contrast, a larger distance between the fast and slow responses indicates a greater residual dependency unexplained by item-difficulty differences. Thus, these items may involve more person and item specificities in the conditional dependence and consequently show a more heterogeneous conditional dependence between the responses and response times.

**Figure 5:**
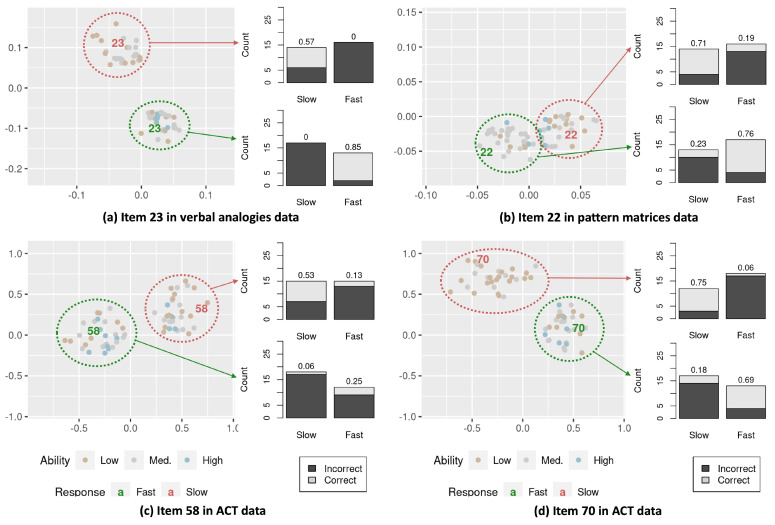
Example items exhibiting heterogeneous item–respondent interactions across slow and fast responses. Two groups of thirty respondents each located near the item positions for slow and fast responses (among those who responded to the item) are displayed in the interaction map. Bar graphs present the proportions of correct items for slow and fast responses calculated from each group.

For a closer inspection, we examine the interaction maps of some example items exhibiting a large distance between the positions of the slow and fast responses (Figure 5). In each interaction map, the locations of the slow and fast responses are shown as red- and green-colored item-number labels, respectively. Two groups of thirty respondents each located near the item positions for the slow and fast responses (among those who responded to the item) are displayed. The bar graphs alongside the interaction map present the proportion of correct responses for the slow and fast responses calculated from each group of respondents. From the bar graphs, we consistently see that the respondents located near the slow-response positions perform disproportionately better on the item when responding slower than faster, whereas those near the fast-response positions perform better when responding faster than slower, irrespective of the overall item-difficulty differences across the slow and fast responses for an item. Such observations imply that the conditional dependence between item responses and response times is heterogeneous across individuals. For instance,

- Item 23 in the verbal analogies data has item-easiness estimates of 1.250 for the slow and 2.930 for the fast responses, indicating that the item is generally easier when responded to faster. However, we observe the opposite pattern for the respondents who are located around the slow-response position in plot (a) of Figure 5; the response accuracy is higher when the response is given slower (proportion correct = .57) than faster (proportion correct = .00).
- The item-easiness estimates for Item 22 in the pattern matrices data are 1.315 and 1.272 for the slow and fast responses, respectively, and .643 and .714 for Item 58 in the ACT data. Although the item-easiness estimates do not have a large difference between the slow and fast responses, we can see from plots (b) and (c) in Figure 5 that the respondents are showing a disproportionately higher correct proportion for the slow or fast responses depending on their distances from the item’s slow- and fast-response positions in the interaction map.
- Item 70 in the ACT data has item-easiness estimates of 1.546 for the slow and 1.103 for the fast responses, suggesting that the item is in general slightly easier when responded to slower. We, however, observe that the respondents located near the fast-response position are performing better when they respond to the item faster than slower.

At the respondent level, the respondents in the high-ability group seem to be more clustered together in Figure 4 compared to the other two groups, suggesting that those respondents with higher abilities generally show less heterogeneity in item–respondent interactions. One interesting observation with the ACT data is that there is an association between respondent positions and their ability levels (see plot (c) of Figure 4). Respondents in the high-ability group tend to be clustered in the lower part of the plot, while the other groups are more spread out in the upper part of the plot. Given this relationship, we can interpret that item positions are associated with respondents’ ability levels. Specifically, it is shown that more difficult items are generally crowded in the lower part of the plot (where the high-ability respondents are clustered), while easier items are scattered in the middle or upper part of the plot (where the low- and medium-ability groups are distributed), especially for fast responses. This can be interpreted as meaning that, for the ACT data, difficult items may tend to be more difficult for low-ability respondents than for high-ability respondents beyond the item and person effects, particularly when the responses are given faster than expected.

There can be several potential sources of the observed association between item positions and respondent-ability levels, which can be examined further to gain insights about item attributes in the test. One possible explanation is that the clustered difficult items may tend to be more effective in differentiating high-ability respondents from others compared to easier items in the chess test. This may result in difficult items being located disproportionately closer to high-ability respondents than to low/medium-ability groups. That is, items’ varying discriminating abilities (unexplained by the model parameters) may have been reflected onto the interaction map. As this pattern is more clearly shown for the fast responses, we may interpret this as that fast responses to these difficult items may have a particularly greater ability to discriminate between high- and low-ability respondents. However, it should also be noted that there can be other potential sources to consider and that it is still unclear how varying item discriminations actually manifest in the interaction map. We plan to examine further on these topics in a separate paper as it is beyond the scope of the current study.

## 6. Conclusions

### 6.1. Summary and Discussion

There recently have been many studies examining conditional dependence between item responses and response times, which have allowed researchers to gain insights beyond what we can learn from the overall correlation between respondents’ ability and speed. However, most previous studies assume that the pattern of residual dependency observed between responses and response times is common for all respondents, disregarding the potential heterogeneity in the pattern across respondents. In this study, we address such heterogeneity by attending to the person and item specificities that may be involved in the conditional dependence between item responses and response times. By applying LSIRT models to item responses classified as slow or fast, we explore the conditional dependence of slow and fast responses and their interactions with respondents.

Our results from three empirical datasets confirm the presence of residual dependency between item responses and response times and further provide evidence that the dependency is heterogeneous across individuals. The average item–respondent distances of items indicate that response accuracy tends to be lower when the response is given slower than expected, and the strength of this association is negatively related to the overall item difficulty. This is consistent with previous studies that revealed a negative conditional dependence between responses and response times (Bolsinova et al. 2017a, 2017c; DiTrapani et al. 2016; Goldhammer et al. 2014; Molenaar and De Boeck 2018; Partchev and De Boeck 2012) and its interaction with item difficulty (Bolsinova et al. 2017a, 2017c; Goldhammer et al. 2014; Meng et al. 2015; Molenaar and De Boeck 2018). At the same time, the interaction map derived after accounting for item differences across slow and fast responses by model parameters demonstrates that the pattern of residual dependency between responses and response times varies across respondents. That is, some respondents may perform better on a certain item (or item types) when responding faster than expected, whereas others may perform worse, irrespective of the general pattern of association between response accuracy and response times. Such findings align with recent observations by Kang et al. (2022b, 2023) that inferred varying conditional dependence between responses and response times across items and persons from the variability in the drift rate and/or starting point of the decision process under diffusion IRT models.

Responses with different speeds are often considered to arise from different response processes or behaviors. For instance, slow and fast responses are known to be associated with automated and controlled response processes, respectively, under the dual-processing framework (Goldhammer et al. 2014; Schneider and Shiffrin 1977; Shiffrin and Schneider 1977). Consistent with previous studies, our results can be interpreted as meaning that easier items are often answered successfully with a fast, automated response process and are prone to slow errors, while more difficult items are likely to be responded to successfully with a slow, controlled response process. It is, however, important to highlight that this tendency can vary across respondents and items, as suggested by the distributions of persons and items in the interaction maps for slow and fast responses in our results. For the data examined in the current study, the heterogeneity appears to be greater for more difficult items, as evidenced by larger distances between the positions of the slow and fast responses for difficult items. This tells us that, for the same difficult item, some respondents can respond more successfully with a controlled process whereas some others may answer more successfully with an automated process.

Through an inspection of the interaction maps, we can attend to such heterogeneity across respondents and items and possibly gain insights into understanding individuals’ response processes for solving an item. We can, for instance, examine which type of response strategy (e.g., a fast automated process or slow controlled process) is expected to be more effective for a respondent to solve specific items or identify respondents who are more prone to making slow or fast errors on certain items or item types. As an example, in the interaction map of the ACT data, a group of difficult items appears to be highly clustered near the high-ability respondents with a large distance from the low- and medium-ability respondents, particularly for fast responses. Considering that slow and fast responses for solving chess problems can be interpreted as stemming from different response strategies (van Harreveld et al. 2007), we can possibly infer from the results that, for these difficult items, the use of a pattern-recognition strategy, known to be faster than a search strategy, is likely to be successful for high-ability respondents while it tends to fail for low-ability respondents. Based on such information, we may not only better understand the underlying cognitive processes for solving items for respective individuals but also possibly provide personalized and detailed diagnostic feedback to the respondents. Furthermore, we may be able to gain a deeper understanding of item attributes or features.

### 6.2. Limitations and Future Studies

Although our study effectively demonstrates the heterogeneity in conditional dependence between responses and response times, it has a limitation in that the relationship is investigated with dichotomized response times (i.e., slow and fast item responses). We think that the dichotomization of response times is one of the simplest and most intuitive approaches for incorporating response time information into the interaction map of the LSIRT model. Although our classification of slow and fast responses based on residual (i.e., double-centered) log-transformed item-response times offers advantages for examining within-individual heterogeneity in response processes across response times, it can be viewed as somewhat arbitrary. While we believe our approach provides robust conclusions, as evidenced by the consistency of our results with previous findings, it may be useful to consider cross-validating the results in future studies if a larger sample is available. Future studies may also consider alternative approaches for defining slow and fast responses such as median split methods (DiTrapani et al. 2016; Partchev and De Boeck 2012) or a stochastic method that attempts to find the best cut-off value for the classification (e.g., Molenaar and De Boeck 2018). It would be interesting to examine whether/how the results change depending on the classification method we choose. Moreover, there have been recent studies illustrating that the residual associations between responses and response times may not be monotone and can be curvilinear (Bolsinova et al. 2017b; Chen et al. 2018; Kang et al. 2022b; Naumann and Goldhammer 2017) . Due to the dichotomization of response times, our approach is not able to address the nonlinearity of the dependence between response accuracy and response times. Future studies might consider applying other approaches such as classifying response times into multiple categories (Bolsinova et al. 2017b) or developing models that incorporate response times as a continuous variable.

Another limitation of this study is that although our approach provides a simple and straightforward way of looking at the heterogeneity of item–person interactions across different response times (which can help to infer respondents’ item-solving process), our approach does not explicitly model or offer an explanation for the cognitive process underlying item-solving behaviors. Other approaches such as process models can be more appropriate and useful for such purposes. For example, diffusion IRT models (Tuerlinckx and De Boeck 2005; Tuerlinckx et al. 2016; van der Maas et al. 2011) that combine the Wiener diffusion model and IRT model can explicitly model the cognitive components of the response processes and possibly provide a better way to explain the cognitive processes involved in item-solving processes (Kang et al. 2022b, 2023).

While we used a Rasch version of the LSIRT model in the current study, it would be interesting to explore whether or how the interaction maps will change if another version of the model (e.g., two-parameter logistic LSIRT) is applied. It is possible that the effect of varying item discriminations is confounded with other potential sources in the interaction map, as potentially shown by the ACT data. Thus, it would be interesting to investigate how the patterns we observe in this study change if we use other versions of the LSIRT model. It is possible that a more general model may provide a more precise way of examining the heterogeneity in conditional dependence between responses and response times as it may account for other possible item effects (including item discriminations). Lastly, we think that the interaction maps can be interpreted in a more meaningful way if more information on items and respondents is available. Future studies might consider exploring data with more detailed information about items and respondents (e.g., item domains, person demographics, etc.) to draw more insightful conclusions from the interaction maps.

## Figures and Tables

**Table 1:** An exemplary illustration of an expanded item-response matrix, treating slow and fast responses to the same item as different items.

Person	Slow Responses				Fast Responses			
	Item 1	Item 2	⋯	Item *I*	Item I+1	Item I+2	⋯	Item I+I
p=1	1	0		NA	NA	NA		1
p=2	0	NA		0	NA	1		NA
p=3	NA	1		NA	1	NA		1
⋮								
p=P	NA	NA		1	0	1		NA

## Data Availability

The original ACT data (van der Maas and Wagenmakers 2005) can be downloaded from https://web.archive.org/web/20051202154523fw_/http:/users.fmg.uva.nl/hvandermaas/chesshtml/act.htm (can be accessed on 10 February 2024). The verbal analogy and pattern matrices data are not publicly available due to confidentiality reasons.
